# Supplementary material for: Curcumin promotes AApoAII amyloidosis and peroxisome proliferation in mice by activating the PPARα signaling pathway
Source: eLife. 2021 Jan 26;10:e63538. doi: 10.7554/eLife.63538 (PMC7880682; doi:10.7554/eLife.63538)
Supplement: Supplementary file 3. [file elife-63538-supp3.docx]

**Supplementary file 3. Specific primers used in real-time qPCR.**

|  | **Foward** | **Reverse** |
| --- | --- | --- |
| *β-actin* | ACAATGAGCTGCGTGTGGCC | CCTCGTAGATGGGCACAGTG |
| *Apoa1* | GTGGCTCTGGTCTTCCTGAC | ACGGTTGAACCCAGAGTGTC |
| *Apoa2* | GCCTGTTCACTCAATACTTTCAG | CAGACTAGTTCCTGCTGACC |
| *Pparα* | GCGTACGGCAATGGCTTTATC | GTTTAGAAGGCCAGGCCGATC |
| *Pparγ* | CCACCAACTTCGGAATCAGCT | GTATTCTTGGAGCTTCAGGTC |
| *Nfκb* | ATGGCAGACGATGATCCCTAC | TGTTGACAGTGGTATTTCTGGTG |
| *Tnfα* | ACGGCATGGATCTCAAAGAC | AGATAGCAAATCGGCTGACG |
| *Il6* | CCCAATTTCCAATGCTCTCC | TGAATTGGATGGTCTTGGTCC |
| *P65* | GCTATAACTCGCCTGGTGAC | CTGTCAGCACCTTAGGAGC |
| *Cd36* | GTGCTGATCCTTTCAGAGTCTC | CAGCAATGAGCCCACAGTTCC |
| *Fabp1* | AGGTCTGCCCGAGGACCTCAT | CCAGTCATGGTCTCCAGTTCG |
| *Scd1* | GAGGCCTGTACGGGATCATA | GCCGTGCCTTGTAAGTTCTGTG |
